# Prevalence and Patterns of Obstructive Sleep Apnea in Asian Indians With Congestive Heart Failure

**DOI:** 10.7759/cureus.11438

**Published:** 2020-11-11

**Authors:** Sukriti Bhalla, Kamal Sharma, R D Yadave, Hardik D Desai, Tanisha Vora, Erum Khan, Purva Shah, Dhigishaba Jadeja, Vishal Bhandari

**Affiliations:** 1 Cardiology, Aakash Healthcare Super Specialty Hospital, New Delhi, IND; 2 Cardiology, U.N. Mehta Institute of Cardiology & Research Centre, Ahmedabad, IND; 3 Cardiology, Sri Balaji Action Medical Institute, New Delhi, IND; 4 Internal Medicine, Gujarat Adani Institute of Medical Sciences, Affiliated With Krantiguru Shyamji Krishna Verma (KSKV) University, Bhuj, IND; 5 Medical Education and Simulation, Smt. Nathiba Hargovandas Lakhmichand (NHL) Municipal Medical College, Ahmedabad, IND; 6 Medicine, Sir Byramjee Jeejeebhoy (BJ) Medical College, Ahmedabad, IND; 7 Interventional Cardiology, Tagore Hospital & Heart Care Centre Private Limited, Jalandhar, IND

**Keywords:** congestive heart failure, obstructive sleep apnea, apnea hypopnea index, asian indian

## Abstract

Background

Sleep-disordered breathing (SDB) has a potential association with the pathogenesis of congestive heart failure (CHF). We assessed the prevalence and patterns of obstructive sleep apnea (OSA) in patients presenting with CHF.

Method

This was a prospective, observational, all-comers study of consecutive 77 confirmed cases of CHF. All these patients were clinically assessed and evaluated for OSA with sleep study after routine blood testing, electrocardiogram (ECG), chest X-ray, and echocardiography.

Results

Of 77 patients with CHF 38 (49.4%) had apnea-hypopnea index (AHI) <5 while 39 (50.6%) had AHI >5. Of these 39, 37 (94.8%) patients showed the clinical features of OSA. The majority (64.9%) of them were males. The majority of OSA (64.9%) had coronary artery disease (CAD) (p<0.05) as the etiology of CHF, followed by dilated cardiomyopathy (32.4%) and valvular heart disease (2.7%). The prevalence of OSA was higher amongst New York Heart Association (NYHA) class 2 (51.4%) as compared to NYHA class 3 (37.8%) and NYHA class 4 (10.8%). There were 12 (32.8%) patients, each having OSA with a heart rate between 71 and 80 bpm and 81 and 90 bpm. Twenty-two (59.5%) had systolic blood pressure (BP) more than 120 mmHg and 20 (54.1%) had diastolic BP more than 80 mmHg. The majority (64.9%) patients had the lowest O_2_ saturation between 80% and 90%. A significantly large number of patients (62.2%) had ejection fraction 21%-30% (p<0.05). The majority (62.16%) of patients with OSA had AHI between 5 and 15. With 5-15 AHI, 20 (87%) patients with OSA had a snoring, tiredness, observed apnea, high BP, BMI, age, neck circumference, and male gender (STOP-Bang) score between 3 and 7 with AHI 5-15 (p<0.05).

Conclusions

In our cohort, the prevalence of OSA in CHF was 50.6%. Predictors of OSA in CHF were left ventricular ejection fraction (LVEF) 20%-30% and NYHA class 2. The majority had AHI between 5 and 15. Sleep apnea screening should be routinely implemented in the evaluation and follow-up of heart failure patients.

## Introduction

Sleep-disordered breathing (SDB) is common in severe congestive heart failure (CHF), which comprises heart failure patients of low left ventricular ejection fraction (LVEF) and is associated with increased morbidity and mortality. Several pathophysiological mechanisms have also been suggested for the development of CHF in patients with obstructive sleep apnea (OSA) such as intermittent hypoxemia, elevated sympathetic activity secondary to recurrent arousals, and baroreflex inhibition. Apart from the etiological association, OSA could also have a higher prevalence in CHF [1-3]. One of the principal disorders with a recognized impact on cardiovascular function and disease is obstructive sleep apnea (OSA). Despite OSA being a risk factor for a cardiovascular disorder, the true prevalence of OSA amongst Asian Indians remains largely unknown, primarily because most people with OSA do not undergo polysomnography and hence may remain undiagnosed.

Obstructive sleep apnea (OSA) is an important risk factor for the development of hypertension, angina pectoris, myocardial infarction, and cor pulmonale. Some recent data suggest that sleep apnea may also lead to the worsening of cardiac dysfunction in patients with CHF especially with heart failure with reduced ejection fraction (HFrEF).

The apnea-hypopnea index (AHI) is the average number of apneic and hypopneic events per hour of sleep, and it is the most common metric to describe the severity of OSA. OSA is said to be present when AHI is >=5 and is considered severe when AHI is >=30, with AHI < 5 ruling out OSA [4]. OSA is strongly associated with obesity, and there is a direct relationship between BMI and the AHI index [5]. OSA is present in over 40% of those with a body mass index (BMI) of 30, and it is especially common in individuals with a BMI of 40.

CHF is a common serious problem in the world [6-8] and despite advances in pharmacotherapy, CHF continues to cause a significant burden of morbidity and mortality [7,9-10]. The prevalence of heart failure is estimated as 2%-3% of the adult population and increases with age [11]. Since SDB is common in patients with CHF, several studies have demonstrated increased mortality in CHF patients suffering from SDB in contrast to those without SDB.

Polysomnography (sleep study) is the gold standard test for the diagnosis of SDB, including OSA [12-13]. Split-night studies, in which the diagnostic study occurs during the first half of the night and continuous positive airway pressure (CPAP) titration occurs during the second half of the night, are increasingly used as a more cost-effective diagnostic-therapeutic strategy.

Targeting a common sleep disorder with treatment not only helps people with heart failure sleep better, but it can also make their hearts healthier. A new study shows that people who suffer from both CHF and OSA can benefit from a nighttime therapy designed to treat the sleep disorder known as CPAP [14].

The aim of this study is to assess the prevalence and patterns of OSA in Asian Indian patients with CHF with low LVEF.

## Materials and methods

This was a prospective, all-comers, observational study of consecutive CHF with reduced LVEF patients over a period of 12 months, with a total of 81 patients of CHF screened, out of which 77 patients completed the study. The study was conducted after obtaining institutional, departmental, and ethics committee clearances. Patients of either gender with symptoms of CHF with low LVEF, age 30-80 years, BMI <25 kg/m^2^, and LVEF <45% were enrolled. Patients with a history of severe hepatic or renal disease, CVA, known case of OSA, malignancy, diabetes, and chronic obstructive pulmonary disease (COPD) were excluded from the study. Patients enrolled were clinically assessed and evaluated for CHF and OSA. Baseline characteristics were noted at the time of recruitment, and pulse and blood pressure measured just prior to polysomnography were captured. All the subjects underwent routine blood testing, electrocardiogram (ECG), chest X-ray, and echocardiography, followed by a sleep study (polysomnography). The snoring, tiredness, observed apnea, high BP, BMI, age, neck circumference, and male gender (STOP-Bang) score was used to correlate the extent of symptoms with AHI and other parameters of the severity of OSA. 

Statistical analysis

The study was statistically analyzed using the Statistical Package for the Social Sciences (SPSS) software version 22 (IBM Corp., Armonk, NY). The qualitative variables were analyzed by the chi-square or Fisher’s exact test and quantitative variables by expressing as mean or median and comparisons with a student’s test.

## Results

Eighty-one patients were screened according to the inclusion criteria in our study, but since four of them could not complete the sleep study, observations were captured and analyzed for the remaining 77 patients. Thirty-eight out of 77 patients (49.4%) had AHI <5 while 39 had AHI >=5.

Table 1 shows the stratified frequency of AHI <5 and >=5 according to age and gender. The majority (35.1%) of OSA patients with AHI >=5 are aged above 70, with 24 (64.9%) males having AHI >=5 and six (75%) males having AHI <5.

**Table 1:** Stratified frequency of AHI <5 and >=5 according to ‘demographics,’ ‘disease and clinical assessment and parameters,’ and ‘STOP-Bang score’ among OSA patients AHI: apnea-hypopnea index, OSA: obstructive sleep apnea, CAD: coronary artery disease, DCMP: dilated cardiomyopathy, NYHA: New York Heart Association, NSR: normal sinus rhythm, LBBB: left bundle branch block, AF: atrial fibrillation, BP: blood pressure, STOP-Bang: snoring, tiredness, observed apnea, high BP, BMI, age, neck circumference, and male gender

Variables	AHI					P-value
	<5			>=5		
	Frequency (n)	%		Frequency OSA (n)	%	
Age group						0.828
<40 yrs	0	0.0%		4	10.8%	
41-50 yrs	1	12.5%		6	16.2%	
51-60 yrs	2	25.0%		9	24.3%	
61-70 yrs	2	25.0%		5	13.5%	
71 - 80 yrs	3	37.5%		13	35.1%	
Sex						
Male	6	75.0%		24	64.9%	0.699
Female	2	25.0%		13	35.1%	
Disease						
CAD	6	75.0%		24	64.9%	0.022
DCMP	0	0.0%		12	32.4%	
VALVULAR	2	25.0%		1	2.7%	
Total	8	100.0%		37	100.0%	
NYHA Class						
II	3	37.5%		19	51.4%	0.355
III	5	62.5%		14	37.8%	
IV	0	0.0%		4	10.8%	
Rhythm						
NSR	6	75.0%		29	78.4%	0.040
AF	0	0.0%		7	18.9%	
LBBB	2	25.0%		1	2.7%	
EF%						
<20	0	0.0%		3	8.1%	0.008
20 - 30	4	50.0%		23	62.2%	
31 - 40	3	37.5%		11	29.7%	
41 - 45	1	12.5%		0	0.0%	
Heart Rate						
<=60	0	0.0%		0	0.0%	0.206
61 - 70	0	0.0%		4	10.8%	
71 - 80	2	25.0%		12	32.4%	
81 - 90	6	75.0%		12	32.4%	
91 - 100	0	0.0%		2	5.4%	
>100	0	0.0%		7	18.9%	
SYSTOLIC BP mmHg						
<=110	2	25.0%		15	40.5%	0.690
111 - 119	0	0.0%		0	0.0%	
>=120	6	75.0%		22	59.5%	
DIASTOLIC BP mmHg						
<=70	6	75.0%		17	45.9%	0.243
71 - 79	0	0.0%		0	0.0%	
>=80	2	25.0%		20	54.1%	
LOWEST O2 SAT %						
70 - 79	0	0.0%		13	35.1%	0.002
80 - 89	6	75.0%		24	64.9%	
90 - 100	2	25.0%		0	0.0%	
STOP-Bang Score						
	0 - 2	0		0.0%	3		8.1%	0.704
	3 - 4	6		75.0%	25		67.6%	
5 - 8	2		25.0%	9	24.3%			
Total	8		100.0%	37	100.0%			

Table 1 shows that the frequency of OSA was found to be the most, i.e. 24 (64.9%), in CAD patients followed by 12 (32.4%) patients with dilated cardiomyopathy (DCMP), and only a single patient with OSA had valvular heart disease. The P-value for the etiology difference amongst OSA in CHF was significant (P < 0.05). More patients were found to have CAD in patients with AHI >=5 amongst CHF.

OSA was a maximum of 19 (51.4%) in NYHA class 2 patients as compared to only 14 (37.8%) patients with NYHA class 3 and 4 (10.8%) patients in NYHA class 4 group. Twenty-nine (78.4%) OSA patients had normal sinus rhythm (NSR), atrial fibrillation (AF) was present in seven (18.9%) OSA patients, and only a single patient had LBBB. There was a significantly greater number of patients with NSR (p<0.05)

Table 1 also shows that a larger number of patients, i.e. 23 (62.2%) with OSA frequency had EF 21%-30% while 11 (29.7%) OSA patients had EF (%) 31-40%. Only three (8.1%) OSA had EF (%) <20 (p<0.05). A significantly greater number of patients were found to have EF (%) between 21 and 30.

Table 1 gives clinical parameters stratified by AHI. It shows that the same number of patients, i.e., 12 in each group (32.4%) with a heart rate between 71 and 80 bpm and 81 and 90 bpm, respectively, had the highest OSA frequency. Twenty-two (59.5%) patients with OSA had systolic BP >=120 while 15 (40.5%) patients had systolic BP <=110 mmHg. 20 patients (54.1%) with OSA had >= 80 mmHg diastolic BP while 17 (45.9%) had diastolic BP <=70. A significant number of patients with OSA had the lowest measured O_2_ saturation of 80%-89% (p<0.05).

Table 1 shows that 25 (67.6%) patients with OSA had a STOP-Bang score of 3-4, nine (24.3%) OSA patients had a STOP-Bang score of 5-8, and only three (8.1%) OSA patients had a STOP-Band score of 0-2.

Our study shows a variation in AHI, i.e. mild (5 to <15), moderate (15 to <30), and severe (>30), therefore, an analysis was done to find the prevalence of OSA in relation to the severity of AHI. The majority, i.e. 23 patients with OSA had AHI between 5 and 15, of which the maximum belonged to the 51-60 year age group. Similarly, males had higher frequencies of OSA (Table 2). Patients with CAD showed maximum OSA in all severity groups followed by DCMP, OSA frequency declined from NYHA class 2 to 4 in AHI 5-15. In AHI 5-15, OSA (65.2%) was most frequent in patients with EF 20-30% (Table 2)

**Table 2:** Stratified frequency of AHI severity according to ‘demographics,’ ‘disease and clinical assessment and parameters,’ and ‘STOP-Bang score’ among OSA patients AHI: apnea-hypopnea index, OSA: obstructive sleep apnea, CAD: coronary artery disease, DCMP: dilated cardiomyopathy, NYHA: New York Heart Association, NSR: normal sinus rhythm, LBBB: left bundle branch block, AF: atrial fibrillation, BP: blood pressure

Variables	AHI Groups			P-value
	5 to <15	15 to <30	>30	
	Frequency OSA (%)	Frequency OSA (%)	Frequency OSA (%)	
Age Groups				0.173
<40 yrs	2 (8.7%)	2 (22.2%)	0 (0%)	
41-50 yrs	4 (17.4%)	2 (22.2%)	0 (0%)	
51-60 yrs	8 (34.8%)	1 (11.1%)	0 (0%)	
61-70 yrs	4 (17.4%)	0 (0%)	1 (20.0%)	
71 - 80 yrs	5 (21.7%)	4 (44.4%)	4 (80.0%)	
Sex				
Male	14 (60.9%)	6 (66.7%)	4 (80.0%)	0.713
Female	9 (39.1%)	3 (33.3%)	1 (20.0%)	
Disease				
CAD	16 (69.6%)	4 (44.4%)	4 (80.0%)	0.330
DCMP	7 (30.4%)	4 (44.4%)	1 (20.0%)	
VALVULAR	0 (0%)	1 (11.1%)	0 (0%)	
NYHA Class				
II	14 (60.9%)	2 (22.2%)	3 (60.0%)	0.312
III	7 (30.4%)	5 (55.6%)	2 (40.0%)	
IV	2 (8.7%)	2 (22.2%)	0 (0%)	
Rhythm				
NSR	19 (82.6%)	6 (66.7%)	4 (80.0%)	0.693
AF	3 (13.0%)	3 (33.3%)	1 (20.0%)	
LBBB	1 (4.3%)	0 (0%)	0 (0%)	
EF%				
<20	2 (8.7%)	1 (11.1%)	0 (0%)	0.703
21 - 30	15 (65.2%)	4 (44.4%)	4 (80.0%)	
31 - 40	6 (26.1%)	4 (44.4%)	1 (20.0%)	
41 - 45	0 (0%)	0 (0%)	0 (0%)	
STOP-Bang Score				
	0 - 2	1 (4.3%)	2 (22.2%)		0 (0%)	0.009
	3 - 7	20 (87.0%)	2 (22.2%)		3 (60.0%)	
5 - 8	2 (8.7%)	5 (55.6%)	2 (40.0%)			
Total	23 (100%)	9 (100%)	5 (100%)			
HR				
	61 - 70	3 (13.0%)	1 (11.1%)		0 (0%)	0.274
	71 - 80	10 (43.5%)	0 (0%)		2 (40.0%)	
81 - 90	7 (30.4%)	3 (33.3%)	2 (40.0%)			
91 - 100	1 (4.3%)	1 (11.1%)	0 (0%)			
>100	2 (8.7%)	4 (44.4%)	1 (20.0%)			
SYSTOLIC BP mmHg				
<=110	11 (47.8%)	3 (33.3%)	1 (20.0%)	0.455
111 - 119	0 (0%)	0 (0%)	0 (0%)	
>=120	12 (52.2%)	6 (66.7%)	4 (80.0%)	
DIASTOLIC BP mmHg				
<=70	11 (47.8%)	5 (55.6%)	1 (20.0%)	0.423
71 - 79	0 (0%)	0 (0%)	0 (0%)	
>=80	12 (52.2%)	4 (44.4%)	4 (80.0%)	
LOWEST O2 SAT %				
70 - 79	6 (26.1%)	2 (22.2%)	5 (100%)	0.005
80 - 89	17 (73.9%)	7 (77.8%)	0 (0.0%)	
90 - 100	0 (0.0%)	0 (0.0%)	0 (0.0%)	

OSA frequency was higher in patients with NSR with heart rates 70-80 bpm and 81-90 bpm with high systolic BP (>=120 mm Hg) and diastolic BP (>=80 mm Hg) though these findings did not reach statistical significance. The highest frequency of OSA patients was seen in those with their lowest oxygen saturation between 80% and 90%. (Table 2).

Table 2 showed that most OSA patients, i.e. 20 (87.0%) had a STOP-Bang score of 3-5 and fell into the category AHI 5 to <15. There were five (55.5%) OSA patients who had a STOP-Bang score of 5-8 and fell in the category AHI 15 to <30 (p<0.05).

## Discussion

In our study, we found that a significant number of patients (39) with CHF with reduced LVEF had AHI value >5. Out of 77 total investigated patients, 45 (58.4%) showed the presence of symptoms of OSA. Thirty-nine (50.6%) had AHI >=5; out of those, 37 (94.8%) showed the presence of the clinical findings of OSA. A high prevalence of SDB amongst the male cohort of CHF with low LVEF was seen in our study; out of 37 patients, OSA was present in 24 males (64.9%) as compared to 13 females (35.1%) (Table 1). These results are consistent with an overall estimation across different countries where the prevalence of SDB is approximately 3% to 7% for adult men and 2% to 5% for adult women in the general population [15].

Javaheri et al. investigated 81 outpatients with stable heart failure, defined as an LVEF of 45%. Most of these patients (75%) had CAD; the remainder had cardiomyopathies. Using an AHI threshold of ≥15/h, the authors found relevant SDB in 41 patients (51%) [16], which is consistent with our data. However, the vast majority of Javaheri's patients had CSA (40%); only 11% had OSA. Although there are important physiological differences between the two, OSA and CSA may often coexist, particularly in patients with heart failure. The results in our study differed and showed that the vast majority of patients had OSA (58.4%) and AHI >=5 in 50.6%.

The results in our study showed that the majority of the patients had NYHA class 2 (Table 1) and had worse LVEF (Table 1). The findings of our study are consistent with a large retrospective analysis of SDB in symptomatic CHF (AHI ≥15/h, NYHA class ≥2, LVEF 27.3±15.6%) by Sin et al. who reported a 61% prevalence of SDB, including CSA in 29% and OSA in 32% [17]. Our findings are similar, where 47% of the patients had OSA and 33% had CSA; patients with OSA belonged to the severity range of 5 to15. 

There was a significant number of patients with NSR, which does not correspond to the results of previous studies [17-18] that have reported a higher incidence of AF. OSA is associated with significant atrial remodeling characterized by atrial enlargement, reduction in voltage, site-specific and widespread conduction abnormalities, and longer sinus node recovery. These features may, in part, explain the association between OSA and AF [19].

Population-based studies have estimated that one in five middle-aged Western adults, with a BMI of 25 to 28 kg/m^2^ have OSA, and one in 20 are symptomatic, with the OSA syndrome [20]. OSA is strongly associated with obesity, and there is a direct relationship between BMI and the AHI index [4]. In our study, OSA was studied in persons with a normal BMI of 18.5-24.9. OSA is highly prevalent in patients with cardiovascular disease and is independently associated with metabolic syndrome in classical symptomatic SDB patients [21]. Therefore, a high body mass index and a trend towards a higher prevalence of diabetes would be expected for CHF with low LVEF patients with OSA [17].

In the present study, OSA has been found to be prevalent in patients with CHF as shown in Table 1. This is consistent with other workers who have reported that with respect to cardiac function, OSA might be a cause of daytime systolic LV dysfunction, which may resolve following effective continuous positive airway pressure (CPAP) therapy [22-25]. Even in adults with normal resting LV function, OSA is associated with an impaired response (increase in cardiac output and stroke volume) to exercise, with CPAP therapy being able to reverse this response. This suggests that pathophysiological changes associated with OSA over the years might progress into structural ventricular damage and lead to symptomatic heart failure. Therefore, OSA might be related to both the causation and progression of heart failure [26]. This is consistent with our findings where the frequency of OSA is more in patients with CHF. So far, it is proven that OSA is an independent risk factor for fatal and non-fatal cardiovascular events [4] and all-cause mortality [27].

Sleep apnea appears to be associated with heart disease regardless of the presence of high blood pressure or other heart risk factors. Studies suggest that patients with moderate-to-severe obstructive sleep apnea have a higher risk of a heart attack.

In the present study, the severity of SDB, when assessed by AHI, in a significant number of OSA patients had the lowest O2 saturation of 80%-90% and belonged to AHI groups 5 to <15 (Table 2). In this context, Sin et al. reported differences in parameters indicative for central and obstructive sleep-disordered breathing events, but the overall mean AHI was not statistically different [17].

Limitations

There are some potential limitations to our study. First, we evaluated SDB by the Special ResMed’s ApneaLink™ Air device [28] and not by complete lab polysomnography. As a consequence, the prevalence and degree of SDB might even be higher than the actual in our cohort of CHF patients. Another limitation may be the fact that the prevalence of SDB in our hospital, which is a tertiary academic cardiac care unit, may be higher than community hospitals. On the other hand, this might also reflect the true prevalence of SDB in symptomatic and severe heart failure. Other studies used patients who were on medication and were obese or overweight, which were not selected for our study. The sample size of our study was also small.

Evidence is lacking to address OSA as a risk factor of CAD or as a prognostic factor of CAD, and more evidence is needed to address CPAP treatment as a useful therapy for patients with CAD and non-symptomatic OSA [29]. However, research in this field is growing rapidly, and data answering some of these questions may soon be available.

## Conclusions

A high prevalence of SDB was found in Asian Indian patients with symptomatic CHF with reduced LVEF, especially amongst males. The prevalence of OSA was highest in CHF patients with LVEF 20%-30% and amongst those with NYHA class I2. A relationship was also found between low EF % and high OSA frequency with AHI >=5. CAD was the most common etiology of CHF, with reduced LVEF in patients who had OSA. The lowest O2 saturation range of 80%-89 % was most frequently observed during the sleep study. Also, a significant number of patients with OSA had the lowest O_2_ saturation of 80%-90% and belonged to AHI 5 to 15 (mild category). More research with bigger sample size is required to find out the association of sleep apnea and CHF, especially with titration studies, and the effect of CPAP treatment on such patients outcomes.
